# A cost-utility and budget impact analysis of allogeneic hematopoietic stem cell transplantation for severe thalassemic patients in Thailand

**DOI:** 10.1186/1472-6963-10-209

**Published:** 2010-07-16

**Authors:** Pattara Leelahavarong, Usa Chaikledkaew, Suradej Hongeng, Vijj Kasemsup, Yoel Lubell, Yot Teerawattananon

**Affiliations:** 1 Health Intervention and Technology Assessment Program (HITAP), 6th Floor, 6th Building, Department of Health, Ministry of Public Health, Tiwanon Road, Muang, Nonthaburi, Thailand; 2 Division of Social and Administrative Pharmacy, Department of Pharmacy, Faculty of Pharmacy, Mahidol University, 447 Sri-Ayudthaya Road, Payathai, Ratchathewi, Bangkok, Thailand; 3 Department of Pediatrics, Faculty of Medicine, Ramathibodi Hospital, Mahidol University, 270 Rama VI Road, Ratchathewi, Bangkok, Thailand; 4 Community Medicine Center, Faculty of Medicine, Ramathibodi Hospital, Mahidol University, 270 Rama VI Road, Ratchathewi, Bangkok, Thailand; 5 Mahidol-Oxford Tropical Medicine Research Unit, Faculty of Tropical Medicine, Mahidol University, 420/6 Ratchawithi Road, Bangkok 10400, Thailand

## Abstract

**Background:**

Hematopoietic stem cell transplantation (HSCT) is the only curative treatment available to severe thalassemic patients. The treatment, however, is very costly, particularly in the context of low and middle income countries, and no studies have been carried out to explore its economic justifiability. This study aimed to estimate the cost-utility of HSCT compared with blood transfusions combined with iron chelating therapy (BT-ICT) for severe thalassemia in Thailand, and to investigate the affordability of HSCT using a budget impact analysis.

**Methods:**

A Markov model was used to estimate the relevant costs and health outcomes over the patients' lifetimes taking a societal perspective as recommended by Thailand's health technology assessment guidelines. All future costs and outcomes were discounted at a rate of 3% per annum. Primary outcomes of interest were lifetime costs, quality adjusted life years (QALYs) gained, and the incremental cost-effectiveness ratio (ICER) in Thai baht (THB) per QALY gained.

**Results:**

Compared to BT-ICT, the incremental cost-effectiveness ratio increased with patient age from 80,700 to 183,000 THB per QALY gained for related HSCT and 209,000 to 953,000 THB per QALY gained for unrelated HSCT among patients aged 1 to 15 years (US$1= 34 THB). The governmental budget impact analysis showed that providing 200 related HSCT to patients aged 1 to 10 years, in accordance with the current infrastructure limitations, would initially require approximately 90 million additional THB per year.

**Conclusions:**

At a societal willingness to pay of 100,000 THB per QALY gained, related HSCT was likely to be a cost-effective and affordable treatment for young children with severe thalassemia in Thailand.

## Background

Thalassemia is the most common gene-related hematological disease in Thailand. With a Thai population of 65 million, approximately 40% carry thalassemia traits and about 1% manifest the disease [1]. The incidence of severe thalassemia (i.e. Hb Bart's hydrops fetalis, β-thalassemia, and β-thalassemia/Hb E) is estimated at 4,253 patients per year [1]. Generally patients with severe thalassemia present with anemia at the first year of life. The provision of regular blood transfusion (BT) is standard practice for the treatment of severe thalassemia. Without ongoing BT, these individuals would have an expected life-span of only a few years. However, provision of BT is hampered by a shortage of blood donations, as well as the high cost of blood screening in order to reduce the residual risk of transmission of blood-borne viruses, including hepatitis and human immunodeficiency virus/acquired immunodeficiency syndrome (HIV/AIDS) [2]. Moreover, BT given over a long period of time can result in iron-overload causing heart failure and damage to other organs associated with high mortality. In order to reduce iron accumulation, iron chelating therapy (ICT) needs to be administered subcutaneously for 8 to 12 hours per day, 5 to 7 days per week. Effective provision of ICT is often compromised by poor compliance as the process itself can have a detrimental effect on quality of life (QoL), especially amongst children [3,4].

Currently, hematopoietic stem cell transplantation (HSCT) is the only curative treatment available to severe thalassemic patients. Hematopoietic stem cells are usually extracted from bone marrow, peripheral blood, and umbilical cord blood. An allogeneic HSCT patient can obtain stem cells from a healthy human leukocyte antigen (HLA)-matched donor, being either a patient's relative (i.e. related HSCT) or from non-related donors (i.e. unrelated HSCT). HLA is determined by conventional serologic typing for class I and II antigen. DNA typing with high-resolution sequence-specific oligonucleotide probes for class I and II loci is undertaken for patients with matched and mismatched HLA-related or mismatched HLA-unrelated donors. Sibling donors are considered ideal as they can inherit identical HLA genes, reducing the probability of graft rejection and other complications. The formula for calculating the chances of a particular person having an HLA-matched sibling is 1 - (0.75)*^n ^*, where *n *denotes the total number of siblings [5]. The average Thai family has two children; therefore only 1 in 4 patients are likely to have an HLA-matched sibling donor [6], while only 3 out of 4 of these potential sibling-donors would themselves be without thalassemia. Thus the proportion of thalassemic patients that would have an HLA-matched sibling donor is approximately 19%. The remainder of the population must rely on unrelated donors. At present there is no local database in place for the identification of such donors, while reliance on foreign databases implies reduced donor availability and an increase in costs.

HSCT procedures are conditioning regimens to eradicate disease and facilitate persistent engraftment [7]. Graft versus host disease (GVHD) prophylaxis is performed to prevent major complications related to severe immune incompetence. All HSCT patients are treated in positive-pressure isolation rooms and receive antibiotics for prevention of *Pneumocystis carinii pneumonia*, Cytomegalovirus, and Ebstein-Barr virus. The criteria of engraftment are an absolute neutrophil count more than 0.5 × 109/L within three consecutive days or a platelet count more than 20 × 109/L without transfusion within seven consecutive days [7-9]. If the patients have engraftment failure after receiving HSCT, a second round of HSCT may be provided to prevent thalassemia recurrence or irreversible aplasia.

As evident, HSCT is a resource-intense procedure requiring high financial expenditure especially at the first year of treatment. Moreover, patients receiving HSCT may experience poor quality of life due to its toxicity and complications. HSCT, however, is the only treatment to cure thalassemia at present, providing patients with longer life expectancy and potentially normal quality of life [6].

In Thailand, healthcare coverage for the provision of HSCT differs amongst the three health insurance schemes. HSCT is provided with full coverage to thalassemic patients who are government employees and their dependents enrolled under the Civil Servant Medical Benefit Scheme (9% of the Thai population) as well as employees enrolled under the Social Security Scheme (11% of the population). Provision of HSCT has not yet been included in the benefit package of the Universal Coverage (UC) scheme that applies to approximately 80% of the Thai population and is managed by the National Health Security Office (NHSO) [10].

An economic evaluation of HSCT for severe thalassemic patients was requested by the NHSO through a topic selection process facilitated by the Health Intervention and Technology Assessment Program (HITAP) [11], the institution responsible for appraising a wide range of health technologies including pharmaceuticals, medical devices, interventions, individual and community health promotion and prevention interventions. HITAP sent out an official letter dated December 27th, 2006 inviting public health agencies at the national level to submit their lists of interventions which they considered to require assessment. The representatives of these fifteen agencies were also invited to participate in a workshop which aimed at prioritizing the proposed health interventions in order to select the top ten most important items for the HITAP assessment process. The economic evaluation of HSCT was ranked as one out of five selected topics in 2007. This cost-utility analysis, therefore, was performed to evaluate and compare the costs and health outcomes for related and unrelated HSCT compared with BT-ICT.

The comparison of related HSCT and BT-ICT is aimed at those patients that have HLA-matched siblings. For the majority of the population however, for whom a matched donor is not readily available, the comparison is between unrelated HSCT and BT-ICT. A preliminary analysis based on the literature showed that when both interventions are available, related HSCT always dominated unrelated HSCT, since in addition to higher costs, the outcome of unrelated HSCT is often compromised by an increase in transplant-related complications including early and late toxicity, mortality and rejection [6,7,11,12]. As a result, there is no head-to-head comparison of related and unrelated HSCT in this analysis, as it is clear that where related HSCT is viable, this would be the preferred option. In instances where HSCT was found to be cost-effective, the impact of including it in the UC package on the government budget was estimated.

## Methods

The lifetime costs and health outcomes for severe thalassemic patients aged 1 to 28 years receiving either HSCT or BT-ICT were compared taking a societal perspective as recommended by the Thailand's health technology assessment guidelines [13]. All future costs and outcomes were discounted at a rate of 3% per annum [14]. Primary outcomes of interest were lifetime costs, quality adjusted life years (QALYs) gained, and the incremental cost-effectiveness ratio (ICER) in Thai baht (THB) per QALY gained. Based on the statement of the Subcommittee for Development of the National List of Essential Drugs and the Subcommittee for Development of the Health Benefit Package and Service Delivery of the NHSO in 2007, the societal willingness to pay (WTP) threshold for a QALY gained for the adoption of health interventions is between 100,000 THB (6,000 PPP$) to 300,000 THB (18,000 PPP$), approximating one to three times per capita Gross Domestic Product (GDP) [15]. These values are in accordance with the recommendations of the Commission on Macroeconomics and Health, World Health Organization, suggesting that health technologies with ICERs below the per capita GDP are considered very cost-effective, those between one and three times per capita GDP being cost-effective, while ICERs above three times per capita GDP indicate that a health technology is not cost-effective [16].

### Economic model

**Figure 1:**
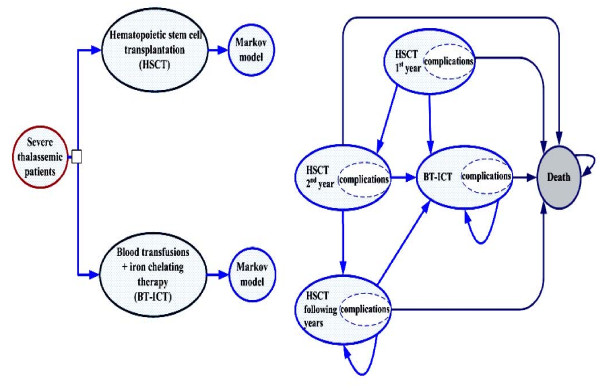
**Schematic diagram of the Markov model**. Each thalassemic patient has two treatment options (i.e. HSCT and BT-ICT). The Markov model consists of five health states and patients receiving HSCT can transition through each of these health states whereas BT-ICT patients can be in either *alive BT-ICT *state or *death *state. The cycle length is one year with a 99-year time horizon. HSCT: hematopoietic stem cell transplantation; BT-ICT: blood transfusion combined with subcutaneous iron chelating therapy.

Figure 1 illustrates the structure of a Markov model used to estimate the relevant costs and health outcomes. The time horizon used is the patients' estimated lifetimes and the length of each cycle is one year. Two mutually exclusive treatment options, related and unrelated HSCT, were compared with BT-ICT (i.e. desferrioxamine--DFO), which is standard practice and currently covered under the UC scheme.

The health states of both related and unrelated HSCT patients consisted of five states with different costs and QoL scores as follows: (i) the first year of HSCT where patients had the highest costs and worst QoL; (ii) the second year of HSCT, where patients had higher costs due to follow up visits and immunosuppressive therapy; (iii) following years after successful HSCT where QoL approximates that of the healthy population and costs were vastly reduced; (iv) HSCT failure resulting in a switch to BT-ICT; and (v) death. Blood transfusions-dependent patients had two health states (i.e. BT-ICT, characterized by low QoL, and the costs of ongoing care and death). The arrows represent the possible transitions from one state to another. Treatment complications were included within all health states as they typically took far less than one year to resolve.

The simulation estimated the costs and health outcomes over a 99-year period to cover the maximum expected lifetime horizon. Based on clinical practice, the following assumptions were used in the model. First, all severe thalassemic patients in this study were assumed to be treated with blood transfusions during the first year of life. Second, ICT was administered via subcutaneous infusion only. Finally, probability of death in HSCT failure patients switching to blood transfusions was similar to that in blood transfusion patients that did not undergo HSCT. All transition probabilities, costs and outcomes variables are shown in Table 1.

**Table 1:** Input parameters used in the model

Parameters	Distribution	Mean	SE	References and type of data
Yearly discount rate (%)				
Costs (range)		3.00 (0-6.00)	-	[14]
Outcomes (range)		3.00 (0-6.00)	-	[14]
Transition probabilities				
*BT-ICT*				
Annual probability of death at age 0-1	Beta	0.010	-	[23]
Annual probability of death at age 2-5	Beta	0.003	-	[23]
Annual probability of death at age 6-10	Beta	0.002	-	[23]
Annual probability of death at age 11-15	Beta	0.010	-	[23]
Annual probability of death at age 16-20	Beta	0.025	-	[23]
Annual probability of death at age 21-30	Beta	0.015	-	[22]
Annual probability of death at age 31 and more	Beta	0.345	-	[21]
*HSCT *				
*Parametric survival: death*				
Constant for baseline hazard	Lognormal	-8.07	2.00	Cohort
Age coefficient for baseline hazard	Lognormal	0.16	0.06	Cohort
Ancillary parameter in Weibull distribution	Lognormal	-0.61	0.41	Cohort
*Parametric survival: failure*				
Constant for baseline hazard	Lognormal	-7.18	1.55	Cohort
Type of HSCT coefficient for baseline hazard	Lognormal	2.60	1.08	Cohort
Ancillary parameter in Weibull distribution	Lognormal	-0.74	0.34	Cohort
Resource cost parameters (THB)				
Total direct medical cost of related HSCT in the 1^st ^year	Gamma	491,985	50,288	Hospital database
Total direct medical cost of related HSCT in the 2^nd ^year	Gamma	42,694	15,535	Hospital database
Total direct medical cost of related HSCT in the following years	Gamma	11,638	3,240	Hospital database
Total direct medical cost of unrelated HSCT at the 1^st ^year	Gamma	735,839	183,560	Hospital database
Total direct medical cost of unrelated HSCT at the 2^nd ^year	Gamma	45,840	20,094	Hospital database
Total direct medical cost of unrelated HSCT in the following years	Gamma	6,385	1,037	Hospital database
Total direct medical cost of BT-ICT per year	Gamma	35,788	4,156	[4]
Total direct non-medical cost of HSCT at the 1^st ^and 2^nd ^year	Gamma	259,994	95,535	Survey
Total direct non-medical cost of BT-ICT and the following year of HSCT	Gamma	37,384	7,040	Survey
Total productivity loss of HSCT in the 1^st ^and 2^nd ^year	Gamma	77,468	70,464	Survey
Total productivity loss of BT-ICT and the following years of HSCT	Gamma	19,171	6,692	Survey
Utility parameters				
Utility of BT-ICT patients	Beta	0.61	0.16	[24,25]
Utility of HSCT patients in first and second year	Beta	0.61	0.16	[24,25]
Utility of HSCT patients from third year on	Beta	0.93	0.05	[26]
				

BT-ICT: blood transfusion combined with subcutaneous iron chelating therapy; HSCT: hematopoietic stem cell transplantation; and THB: Thai baht in 2008 value.

### Cost variables

All costs were converted and reported in 2008 THB (US$1= 34 THB) using the consumer price index (CPI) [17]. For international comparison, costs were converted to international dollars using a purchasing power parity (PPP) exchange rate (1 PPP$(2008) = 15.954 THB) [18]. Direct medical costs were obtained from two data sources. First, the costs for blood transfusion-dependent patients were obtained from a cost analysis by Torcharus et al [4]. The study population consisted of 124 severe thalassemia patients. Mean direct medical costs per year were 35,788 THB (SE = 4,156). The costs of patients receiving HSCT were retrieved from a hospital database at a teaching hospital between 1989 and 2007. The charges per patient per year were adjusted using a cost-to-charge ratio of 0.8 [19].

Both direct non-medical and indirect costs were collected by interviewing severe thalassemic patients and their caregivers at the teaching hospital after informed consent was obtained. Ethical approval for this study was granted by the Committee on Human Rights Related to Research Involving Human Subjects, Mahidol University for cost and clinical data collection. Two HSCT patients and twenty eight BT-ICT patients were interviewed by questionnaire. Mean age was 18 years (Range = 1-47 years). Direct non-medical costs including costs of transportation, meals, accommodation, facilities, and informal care were 37,384 THB (SE = 7,040) for BT-ICT patients and 259,994 THB (SE = 95,535) at the first and second year of HSCT [13]. Indirect costs referred to productivity losses due to sick leave and were estimated at 19,171 THB (SE = 6,692) for BT-ICT and 77,468 THB (SE = 70,464) at the first and second year of HSCT [13]. Since there were no data available on direct non-medical and indirect costs of HSCT patients after the first two years of treatment, it was assumed that these costs of HSCT patients were similar to those receiving BT-ICT in the following years. Resource cost parameters are presented in Table 1.

### Clinical variables

Transitional probabilities or *tp(u) *(i.e. transition to failure and transition to death) were required for the Markov model to simulate patients with different ages when starting HSCT. First, patient-level time to event data with clinical variables such as survival time, demographic characteristics, and patient status (alive/dead) were collected from the medical records of 67 patients receiving either related (44) or unrelated HSCT (23) at the teaching hospital between 1989 and 2007. There were 26 cases with β- thalassemia and 41 cases with β-thalassemia/HbE. Mean age was 8 years (Range = 1-28 years). These patients were classified as moderate to high risk according to Lucarelli or Pesaro classification [6]. Second, a parametric survival-time model using Weibull regression was applied in order to yield *H(t) *which is the cumulative hazard, λ (lambda) which is the scale parameter, and γ (gamma) which is the shape parameter based on the below survival function (see equations 1-3). The survival function, *S(t) *which describes the probability of survival as a function of patient age at the start of HSCT is [20]:

(1) S(t)=exp{−H(t)}

(2) $$H\left(t\right)=\lambda t^{\gamma }$$

where *H(t) *is the cumulative hazard; λ (lambda) is the scale parameter; *t *is time in days; and ancillary or γ (gamma) is the shape parameter that describes the instantaneous hazard rate *h(t)*, which increases with age at the start of HSCT if γ is more than one. The influence of patient age at the start of HSCT and whether they had a related or unrelated donor on mortality and treatment failure was assessed and found that λ for failure event depended on the type of donor and λ for death event depended on the age covariate as illustrated in the following formula:

(3) λ=exp{constant+(age coefficient*age at the start of treatment)}

Last, the transitional probability of the event of interest during the cycle, *tp(u)*, is calculated using *H(t-u) *and *H(t) *as well as being estimated from the following formula (where *u *is the cycle length of the model):

(4) tp(u)=1−exp{H(t−u)−H(t)}

In addition, for patients receiving BT-ICT, the transition probabilities converted to annual probabilities of death were derived from available cohort studies [21-23]. Based on the cohort of 67 patients, no one received HSCT more than twice. Therefore, in the model it was assumed that if the HSCT patients had failed twice, patients would receive BT-ICT for the rest of their lives.

### Quality of life variables

Utility measures for patients' quality of life were obtained from a systematic review of electronic databases. Two databases (PubMed and Center of Reviews and Dissemination) were searched using the following keywords: ("Quality of Life"[Mesh] OR "Quality-Adjusted Life Years"[Mesh] OR "Models, Economic"[Mesh]) AND ("Thalassemia"[Mesh] OR "beta-Thalassemia"[Mesh] OR thalassaemia) and (thalassemia OR thalassaemia) AND "quality of life", respectively. Inclusion criteria were where QoL is presented in a utility index (0=death and 1=full health) and measured by time trade-off (TTO), standard gamble (SG), or EQ-5D instruments. Two eligible studies reported that the mean utility indices of patients receiving BT-ICT were 0.61 and 0.66 [24,25]. A Bayesian random effects meta-analysis using WinBUGS1.4 (Medical Research Council and Imperial College of Science, Technology and Medicine, United Kingdom) was used to obtain the pooled estimate of 0.61 (SE = 0.16). No studies relating to the QoL of severe thalassemic patients receiving HSCT were identified, therefore the utility of thalassemic patients receiving HSCT at the first and second years was assumed to be the same as that of thalassemic patients receiving BT-ICT for the worst case analysis. The utility of HSCT patients in the subsequent years was derived from the utility of HSCT patients in other diseases following HSCT (i.e. acute myeloid leukemia, multiple myeloma, non-Hodgkin lymphoma, and Hodgkin lymphoma) equal to 0.93 (SE 0.05) [26].

### Uncertainty analysis

One-way sensitivity analysis was performed to examine the uncertainty surrounding each parameter individually and results were presented using a tornado diagram. In addition, a probabilistic sensitivity analysis (PSA) was conducted to examine the effect of all parameter uncertainty simultaneously using a second order Monte Carlo simulation performed by Microsoft Excel 2003 (Microsoft Corp., Redmond, WA) [20]. Probability distributions were assigned as follows [27]: (i) beta-distributions were assigned where parameter values ranged between zero and one, such as in probability and utility parameters, (ii) gamma-distributions were specified when parameter values were above zero and positively skewed, such as in costs, and (iii) a log-normal distribution was used for survival parameters. The mean, SE, and distribution of input parameters used in the model are shown in Table 1. A Monte Carlo simulation was run for 1,000 iterations to yield a range of probable values for total costs, health outcomes, and ICERs. In addition, the maximum expected net monetary benefit (NMB) was calculated for each ceiling ratio value (the value society would be WTP for a QALY gained). Results of the PSA were presented as cost-effectiveness acceptability curves.

### Budget impact analysis

A Markov-based budget impact model was developed to evaluate direct medical costs for severe thalassemic patients based on a governmental perspective over 15 fiscal years. The model compared direct medical costs for BT-ICT to those of HSCT, where this was found to be a cost-effective option at a ceiling ratio of 100,000 THB per QALY gained [15], approximating the Thai GDP per capita [18]. The actual number of additional HSCT procedures that could be carried out in practice is currently restricted to approximately 200 thalassemic patients as there are only four university hospitals that have the necessary specialists and suitable infrastructure to carry out HSCT (Suradej Hongeng, Vijj Kasemsup, Ramathibodi University Hospital, oral communication, March 24, 2009). Thus, the actual budget impact on the universal health insurance scheme was estimated for the maximum expected number of severe thalassemic patients that could receive HSCT at the four teaching hospitals, rather than the incidence of severe thalassemia.

## Results

### Cost-utility analysis

**Figure 2:**
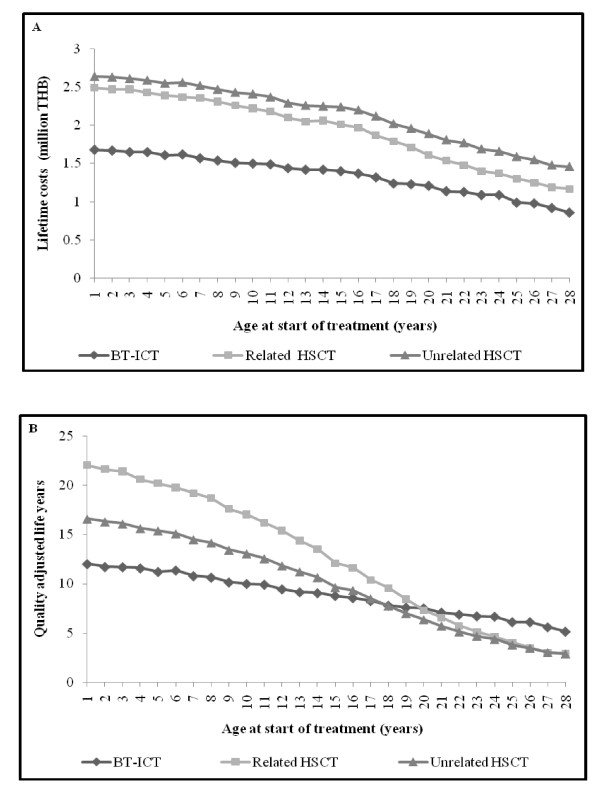
**Lifetime costs and quality adjusted life years of treatment**. (A) The lifetime costs and (B) Quality adjusted life years of treatment options of severe thalassemia classified by patient age at the start of treatments. THB: Thai baht (in 2008 value); BT-ICT: blood transfusion combined with subcutaneous iron chelating therapy; and HSCT: hematopoietic stem cell transplantation.

Average lifetime costs and QALYs gained of related HSCT and unrelated HSCT compared with BT-ICT classified by patient age at the start of treatment are shown in Figure 2. The lifetime costs were the highest for unrelated HSCT, followed by related HSCT, while BT-ICT incurred the lowest cost across all age groups. Both related and unrelated HSCT yielded more QALYs than BT-ICT amongst patients aged 1 to 19 and 1 to 17 years, respectively, after which BT-ICT yielded more QALYs.

When the costs and QALYs of related HSCT and unrelated HSCT were compared, it was confirmed that related HSCT always dominated unrelated HSCT due primarily to an increase in transplant-related complications including early and late toxicity, mortality and rejection [6,7,11,12]. Thus, for patients who had HLA-matched siblings the ICER of related HSCT was compared with that of BT-ICT (Table 2), while for those who did not have an HLA-matched sibling, the ICER of unrelated HSCT was compared with that of BT-ICT (Table 3), with no direct comparison required between related and unrelated HSCT. The ICERs of both related and unrelated HSCT increased with patient age up to the age of 20 and 18 years, respectively, at which point they were both dominated by BT-ICT which incurred lower cost and yielded more QALYs. The ICERs of related HSCT for patients aged 1 to 19 years were 80,700 to 574,000 THB per QALY gained, and the ICERs of unrelated HSCT for patients aged 1 to 17 years were 209,000 to 3,270,000 THB per QALY gained.

**Table 2:** ICER of related HSCT compared to BT-ICT, classified by patient age

Age (year)	Incremental cost	Incremental QALY	ICERs of related HSCT compared to BT-ICT
	
	million THB	QALY gained	THB per QALY gained*
1	0.81	10.00	80,700
5	0.78	8.96	86,800
10	0.72	7.02	103,000
15	0.61	3.32	183,000
17	0.55	2.14	257,000
18	0.55	1.78	308,000
19	0.48	0.84	574,000
20	0.40	-0.20	Dominated^§^
25	0.30	-2.05	Dominated^§^
28	0.31	-2.17	Dominated^§^

ICER: incremental cost-effectiveness ratio; HSCT: hematopoietic stem cell transplantation; BT-ICT: blood transfusion combined with subcutaneous iron chelating therapy; THB: Thai baht (in 2008 value); and QALY: quality adjusted life year.

*ICERs are rounded up to nearest 1,000 THB.

^§^Negative ICER due to higher effectiveness and lower costs of BT-ICT compared with HSCT.

**Table 3:** ICER of unrelated HSCT compared to BT-ICT, classified by patient age

Age (year)	Incremental cost	Incremental QALY	ICER of unrelated HSCT compared to BT-ICT
	
	million THB	QALY gained	THB per QALY gained*
1	0.96	4.57	209,000
5	0.94	4.16	225,000
10	0.91	3.05	297,000
15	0.84	0.87	953,000
17	0.80	0.26	3,270,000
18	0.78	-0.01	Dominated^§^
19	0.73	-0.57	Dominated^§^
20	0.68	-1.12	Dominated^§^
25	0.59	-2.28	Dominated^§^
28	0.60	-2.22	Dominated^§^

ICER: incremental cost-effectiveness ratio; HSCT: hematopoietic stem cell transplantation; BT-ICT: blood transfusion combined with subcutaneous iron chelating therapy; THB: Thai baht (in 2008 value); and QALY: quality adjusted life year.

*ICERs are rounded up to nearest 1,000 THB.

^§^Negative ICER due to higher effectiveness and lower costs of BT-ICT compared with HSCT.

### Uncertainty analysis

**Figure 3:**
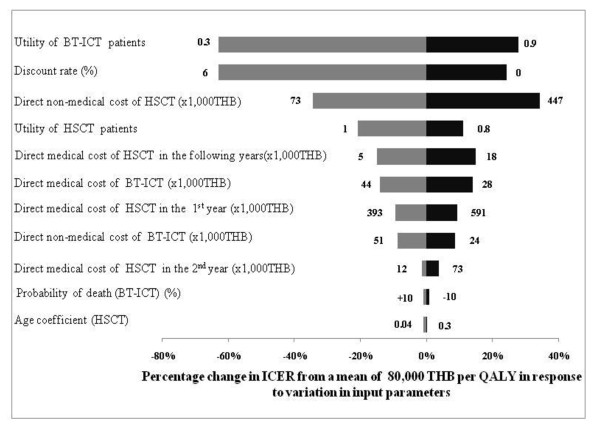
**Tornado diagram**. The diagram shows the percentage change in the ICER attributable to the change of each individual parameter. The numbers at each end of the bars indicate the most extreme values used in the sensitivity analysis. ICER: incremental cost-effectiveness ratio; THB: Thai baht (in 2008 value); QALY: quality adjusted life year; HSCT: hematopoietic stem cell transplantation; and BT-ICT: blood transfusion combined with subcutaneous iron chelating therapy.

Figure 3 shows a tornado diagram presenting the results of one-way sensitivity analyses in the case of patients at 1 year of age receiving related HSCT. It was found that when altering the value of each parameter within plausible ranges, the ICER per QALY gained was most sensitive to changes in the utility of blood transfusion patients, followed by changes in the discount rate to 0% and 6% per annum, direct non-medical costs of related HSCT, utility of HSCT patients, and direct medical costs. It is noteworthy that the ICER was less sensitive to changes in the transition probabilities of both related HSCT and BT-ICT.

**Figure 4:**
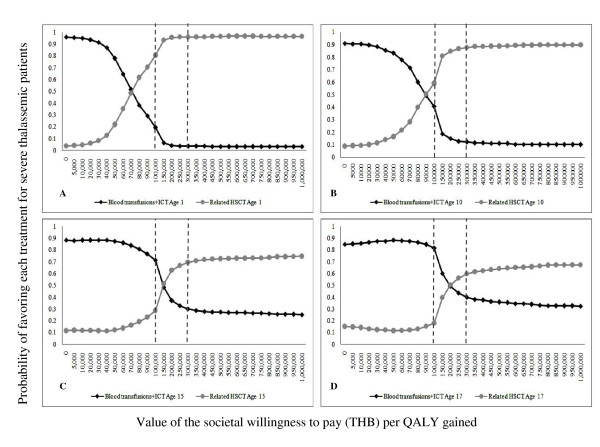
**Cost-effectiveness acceptability curve for related HSCT compared with BT-ICT**. These graphs demonstrate the probabilities of each intervention being cost-effective at different ceiling ratios, classified by age (year) at the start of treatment. (A) Patient aged 1 year, (B) Patient aged 10 years, (C) Patient aged 15 years, and (D) Patient aged 17 years. Dashed lines represent the willingness to pay thresholds for the adoption of health interventions in Thailand. BT-ICT: blood transfusion combined with subcutaneous iron chelating therapy; HSCT: hematopoietic stem cell transplantation; QALY: quality adjusted life year; and THB: Thai baht.

Figure 4 illustrates the cost-effectiveness acceptability curves based on the PSA results for related HSCT classified by patient age at the beginning of treatment. The vertical dashed lines show the WTP thresholds of 100,000 and 300,000 THB per QALY gained. At a WTP threshold of 100,000 THB per QALY gained, the probabilities that related HSCT would be cost-effective were 81%, 59%, 29%, and 18% for patients aged 1, 10, 15, and 17 years, respectively. At a WTP threshold of 300,000 THB per QALY gained, the probabilities that related HSCT would be cost-effective were 96%, 88%, 70%, and 60% for patient aged 1, 10, 15, and 17 years, respectively.

**Figure 5:**
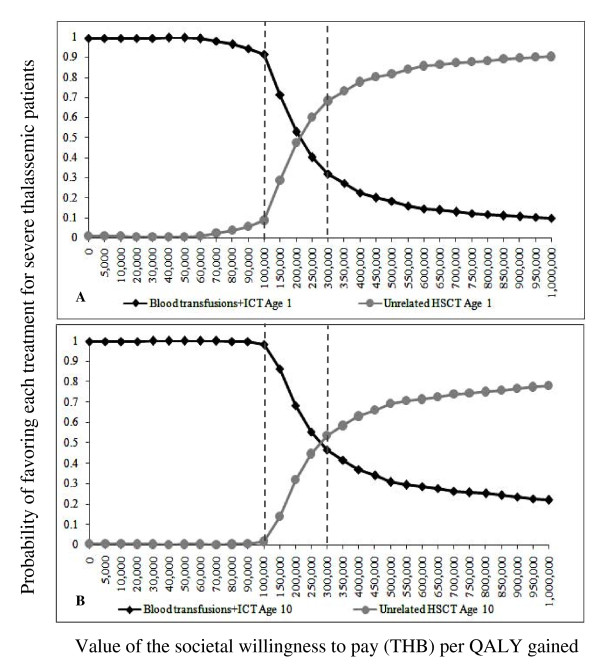
**Cost-effectiveness acceptability curve for unrelated HSCT compared with BT-ICT**. These graphs demonstrate the probabilities of each intervention being cost-effective at different ceiling ratios, classified by age (year) at the start of treatment. (A) Patient aged 1 year, and (B) Patient aged 10 years. Dashed lines represent the willingness to pay thresholds for the adoption of health interventions in Thailand. BT-ICT: blood transfusion combined with subcutaneous iron chelating therapy; HSCT: hematopoietic stem cell transplantation; QALY: quality adjusted life year; and THB: Thai baht.

Figure 5 shows the cost-effectiveness acceptability curves based on the PSA results for unrelated HSCT classified by patient age at the beginning of treatment. At a WTP threshold of 100,000 THB per QALY gained, unrelated HSCT would not be cost-effective when compared with BT-ICT, as the ICER is higher than the ceiling ratio. At a WTP threshold of 300,000 THB per QALY gained, the probabilities that unrelated HSCT would be cost-effective were 68% and 54% for patient aged 1 and 10 years, respectively.

### Budget impact analysis

The budget impact analysis shows how the implementation of related HSCT in patients for whom it was found to be cost-effective at a WTP threshold of 100,000 THB per QALY gained would impact future expenditure [15], should the NHSO decide to include HSCT in the benefit package of the UC scheme. The number of treatments for which this is calculated is 200 thalassemic patients per year (Suradej Hongeng, Vijj Kasemsup, Ramathibodi University Hospital, oral communication, March 24, 2009), due to infrastructure restrictions, and their impact on the government budget during fiscal years 2008 to 2022 is presented in Table 4.

**Table 4:** Estimated budget impact during fiscal years 2008 to 2022 of provision of HSCT to 200 severe thalassemic patients (aged 1-10) per year

Estimated budget impact (million THB)			Incremental budget
	
Fiscal year	BT-ICT	Related HSCT	
2008	7	98	91
2009	14	104	90
2010	20	103	83
2011	26	102	76
2012	32	101	69
2013	37	100	63
2014	42	99	57
2015	46	99	53
2016	50	98	48
2017	54	97	43
2018	58	96	38
2019	61	95	34
2020	64	93	29
2021	67	92	25
2022	69	91	22
**Total**	**647**	**1,468**	**821**

BT-ICT: blood transfusion combined with subcutaneous iron chelating therapy; HSCT: hematopoietic stem cell transplantation; and THB: Thai baht (in 2008 value).

The costs shown in the table related to a cohort of patients that increases by 200 each year (minus fatalities), where in the BT-ICT column the cost consists of their ongoing treatments, while in the HSCT column the costs comprise of 200 treatments each year, the cost of BT-ICT for treatment failures, and the cost of follow-up procedures. It should be noted that the follow up costs were significantly reduced over time owing to the effect of discounting and reduction of costs of long-term care for HSCT patients. The rightmost column shows the actual impact of the government budget. Notwithstanding future possible changes in HSCT and BT-ICT costs, as the number of patients receiving BT-ICT increases continuously the difference in cost gradually decreases and over a longer time span would eventually become negative (i.e. HSCT would become the more affordable option).

## Discussion

This study is the first to compare the cost-effectiveness of related and unrelated HSCT to BT-ICT for severe thalassemic patients. Based on the WTP threshold of 100,000 THB per QALY gained [15,28], this analysis shows that provision of HSCT to severe thalassemic patients with related or sibling donors was likely to be cost-effective only when provided to patients aged up to 10 years. Although we found that providing related HSCT to the youngest patients yields the maximum benefit, there are ethical and practical issues that require further consideration. For example, the availability of a sibling donor for a one year old patient will be more restricted than those for ten year old patients, as it is more likely that the 10 year old patients would have healthy siblings (having both elder and younger siblings that could act as donors). The majority of severe thalassemic patients, however, do not have the option of related HSCT, since most of them are either single children or they have no HLA-matched non-thalassemic relative or sibling donor. For these patients unrelated HSCT was the only alternative to BT-ICT. Although at a WTP of 300,000 THB per QALY gained unrelated HSCT was likely to be considered a cost-effective option for patients aged up to 10 years, it might not yet be appropriate within the Thai context, where the identification procedure for HLA-matched donors is very limited and expensive, as donors have to be identified from foreign sources such as the Tzu chi Taiwan Marrow Donor Registry [8]. Furthermore, if unrelated HSCT procedure failed, the patients would be more likely to switch to standard therapy (BT-ICT) in the absence of another donor. The provision of HSCT only to younger patients for whom sibling donors are available, however, raises a substantial equity concern in that patients without a sibling donor or over the age of 10 would not be eligible to benefit from a potentially life-saving treatment.

The governmental budget impact demonstrated that the provision of related HSCT to patients for whom HSCT was found to be cost-effective could eventually result in lower government expenditure than ongoing BT-ICT. The budget impact analysis took a very practical perspective in calculating the costs for only 200 patients per year in accordance with the current available infrastructure and human resources, especially hematologists. If the Thai government were to provide additional budget to significantly expand HSCT services, it is likely that the cost-per case would fall due to economies of scale, improving the cost-effectiveness of HSCT. Such a scenario was considered unlikely in the near future therefore to ensure the relevance of the analysis to current policy making needs, the restriction to 200 patients a year was left in place. This restriction raises further equity concerns in deciding how to allocate these treatments.

Previous published studies suggested that allogeneic stem cell transplantation was more effective amongst younger severe thalassemic patients, as older patients had poorer outcomes and higher transplant-related mortality [29,30]. This could be explained by the fact that older thalassemic patients had more progressive disease and prolonged iron overload having received blood transfusions for a longer period of time [6]. This study also showed that the QALY gained among younger HSCT patients was higher than older ones.

This study had several limitations. First, due to the lack of survival data for patients treated with blood transfusions in Thailand, their transition probabilities were based on survival analysis studies in Iran [21-23]. The prevalence of thalassemia and its treatment in Iran were found to be similar to that of Thailand, in that severe thalassemia in Iran was the most prevalent genetic disease and its standard therapy was the provision of BT-ICT. Likewise, the survival data and transition probabilities for HSCT patients were obtained from a relatively small cohort; this area is a priority for future research. Second, the direct medical costs of the interventions were obtained from different sources. While both sources originate in the Thai context, the costs of blood transfusions were derived from a cost of illness study in three treatment centers in Thailand [4] whereas the costs of HSCT were estimated from a hospital database. Using a single hospital database could however underestimate the true costs of blood transfusions, as patients might receive these in a number of different hospitals. HSCT patients on the other hand, need to be followed up regularly at the same hospital so all costs could be collected in a single hospital database. Third, the sensitivity analysis indicated that the ICER per QALY gained was most sensitive to changes in the utility of BT-ICT patients which this study obtained from foreign data (i.e. Australia and UK). This is identified as an area where further studies using local data are needed. Fourth, no estimates were available for the utility parameter of patients receiving HSCT, so that the utility of HSCT patients with malignant diseases was used instead [26]. Lastly, the utilities for all HSCT states were similar even though previous studies revealed that the QoL in the early post HSCT states would be poor due to possible complications (e.g. graft-versus-host-disease, infection, and graft failure); in subsequent states QoL was closer to the norm and better than patients receiving blood transfusions [6,30-32].

## Conclusions

The results of this study were twice presented to the Subcommittee for Development of the Health Benefit Package and Service Delivery of the NHSO. Although the analysis found that related HSCT for patients aged less than 10 years was the most cost-effective option in the Thai context, the currently limited infrastructure implies that this will only be available to a minority of patients, proving to be a major obstacle to policy formulation and implementation. This is indicative of a broader problem that is particularly acute in low and middle income countries, where life-saving and cost-effective technologies are becoming more readily accessible while the infrastructure and financial resources are not yet available to provide these on a large scale and in an equitable manner. There are immense challenges to rationing such services in deciding whether these should be allocated based on a "first come first serve", "severity of disease", "fair inning" or a "lottery principle" [33]. As a result, the Subcommittee has not reached a consensus and provided any policy recommendations to the NHSO. This situation reiterates that economic analysis alone is insufficient in providing practical decision recommendations to policy makers where such pertinent equity concerns are present. There is an urgent need to carefully consider social, ethical and moral dimensions of this health technology beyond its immediate economic benefits.

## Competing interests

The authors declare that they have no competing interests.

## Authors' contributions

PL performed the research, analyzed data, and drafted the manuscript. UC performed the research and drafted the manuscript. SH and VK participated in clinical research part. YL participated in its design and drafted the manuscript. YT designed the research and drafted the manuscript. All authors read and approved the final manuscript.

## Pre-publication history

The pre-publication history for this paper can be accessed here:

http://www.biomedcentral.com/1472-6963/10/209/prepub

## References

[B1] Tienthavorn V Patrakulvanish S Pattanapongthorn J Voramongkol N Sangoarnsermsri T Charoenkwan P Prevalence of thalassemia carrier and risk of spouse to have a severe thalassemic child in Thailand National Conference on Thalassemia 11st; Miracle Grand Hotel, Bangkok 2005 Department of Health, Ministry of Public Health, Thalassemia Foundation of Thailand

[B2] Bunyadharokul S Budget impact of the thalassemia management under the National Health Security Scheme Master's thesis. Mahidol, Pharmacy 2008

[B3] Mahityutthana J Health-related quality of life and satisfaction with health service of thalassemia patients 2007 Master's thesis. Mahidol University, Faculty of Pharmacy

[B4] Torcharus K Nuchprayoon I Indaratna K Riewpaiboon A Thawornshareansuk M Cost of illness, satisfaction and health related quality of life of thalassemia patients 2006 Nonthaburi: Clinical Research Collaboration Network

[B5] Armitage JO Bone Marrow Transplantation N Engl J Med 1994 330 12 827 838 10.1056/NEJM199403243301206 8114836

[B6] Lucarelli G Gaziev J Advances in the allogeneic transplantation for thalassemia Blood Rev 2008 22 2 53 63 10.1016/j.blre.2007.10.001 18039551

[B7] Hongeng S Pakakasama S Chuansumrit A Sirachainan N Kitpoka P Udomsubpayakul U Ungkanont A Jootar S Outcomes of transplantation with related- and unrelated-donor stem cells in children with severe thalassemia Biol Blood Marrow Transplant 2006 12 6 683 687 10.1016/j.bbmt.2006.02.008 16737942

[B8] Hongeng S Pakakasama S Chaisiripoomkere W Chuansumrit A Sirachainan N Ungkanont A Jootar S Outcome of transplantation with unrelated donor bone marrow in children with severe thalassaemia Bone Marrow Transplant 2004 33 4 377 379 10.1038/sj.bmt.1704361 14676781

[B9] Hongeng S Pakakasama S Chuansumrit A Sirachainan N Sura T Ungkanont A Chuncharunee S Jootar S Issaragisil S Reduced intensity stem cell transplantation for treatment of class 3 Lucarelli severe thalassemia patients Am J Hematol 2007 82 12 1095 1098 10.1002/ajh.21002 17674372

[B10] Teerawattananon Y Mugford M Tangcharoensathien V Economic evaluation of palliative management versus peritoneal dialysis and hemodialysis for end-stage renal disease: evidence for coverage decisions in Thailand Value Health 2007 10 1 61 72 10.1111/j.1524-4733.2006.00145.x 17261117

[B11] Davies S Kollman C Anasetti C Engraftment and survival after unrelated-donor bone marrow transplantation: a report from the National Marrow Donor Program Blood 2000 96 4096 4102 11110679

[B12] La Nasa G Giardini C Argiolu F Locatelli F Arras M De Stefano P Ledda A Pizzati A Sanna MA Vacca A Unrelated donor bone marrow transplantation for thalassemia: the effect of extended haplotypes Blood 2002 99 12 4350 4356 10.1182/blood.V99.12.4350 12036861

[B13] Riewpaiboon A Measurement of costs J Med Assoc Thai 2008 91 suppl 2 S28 37 19253485

[B14] Permsuwan U Guntawongwan K Buddhawongsa P Handling time in economic evaluation studies J Med Assoc Thai 2008 91 suppl 2 S53 58 19255986

[B15] The Subcommittee for Development of the National List of Essential Medicines [The threshold at which an intervention becomes cost-effective Meeting of the Subcommittee for Development of the National List of Essential Medicine 9/2007] Dec 20; Jainad Narendhorn meeting room, Food and Drug Administration, Ministry of Public Health Thailand 2007

[B16] The Commission on Macroeconomics and Health Macroeconomics and Health: Investing in Health for Economic Developement 2002 Geneva: World Health Organization

[B17] Ministry of Commerce Thailand. Report for Consumer Price Index of Thailand year 2000-2008. [2008 July 9] http://www.indexpr.moc.go.th/price_present/cpi/data/index_47.asp?list_month=07&list_year=2551&list_region=country

[B18] International Monetary Fund. The World Economic Outlook Database Washington, DC: IMF Publication Services http://www.imf.org/external/pubs/ft/weo/2008/01/weodata/index.aspx [updated April 2008December 18,2008]

[B19] Ministry of Public Health Reimbursement rate of public health facilities 2004 Nonthaburi: Ministry of Public Health

[B20] Briggs A Sculpher M Claxton K Decision modelling for health economic evaluation 2006 Oxford: Oxford University Press

[B21] Yavarian MD Farsheedfar G Karimi M Almoazzez M Harteveld C Giordano P Survival analysis of transfusion dependent beta-thalassemia major patients J Res Health Sci 2006 6 1 8 13

[B22] Kosaryan M Vahidshahi K Karami H Forootan MA Ahangari M Survival of thalassemic patients referred to the Boo Ali Sina Teaching Hospital, Sari, Iran Hemoglobin 2007 31 4 453 462 10.1080/03630260701641294 17994379

[B23] Roudbari M Soltani-Rad M Roudbari S The survival analysis of beta thalassemia major patients in South East of Iran Saudi Med J 2008 29 7 1031 1035 18626536

[B24] Osborne RH De Abreu Lourenco R Dalton A Houltram J Dowton D Joshua DE Lindeman R Ho PJ Quality of life related to oral versus subcutaneous iron chelation: a time trade-off study Value Health 2007 10 6 451 456 10.1111/j.1524-4733.2007.00200.x 17970927

[B25] Karnon J Tolley K Oyee J Jewitt K Ossa D Akehurst R Cost-utility analysis of deferasirox compared to standard therapy with desferrioxamine for patients requiring iron chelation therapy in the United Kingdom Curr Med Res Opin 2008 24 6 1609 1621 10.1185/03007990802077442 18439348

[B26] Slovacek L Slovackova B Jebavy L Global quality of life in patients who have undergone the hematopoietic stem cell transplantation: finding from transversal and retrospective study Exp Oncol 2005 27 3 238 242 16244589

[B27] Limwattananon S Handling uncertainty of the economic evaluation result: sensitivity analysis J Med Assoc Thai 2008 91 suppl 2 S59 S65 19253488

[B28] International Monetary Fund. The World Economic Outlook Database Washington, DC: IMF Publication Services http://www.imf.org/external/pubs/ft/weo/2008/01/weodata/index.aspx [2008 December 18]

[B29] Gaziev J Sodani P Polchi P Andreani M Lucarelli G Bone marrow transplantation in adults with thalassemia: Treatment and long-term follow-up Ann N Y Acad Sci 2005 1054 196 205 10.1196/annals.1345.024 16339666

[B30] Lawson SE Roberts IA Amrolia P Dokal I Szydlo R Darbyshire PJ Bone marrow transplantation for beta-thalassaemia major: the UK experience in two paediatric centres Br J Haematol 2003 120 2 289 295 10.1046/j.1365-2141.2003.04065.x 12542489

[B31] Cheuk DK Mok AS Lee AC Chiang AK Ha SY Lau YL Chan GC Quality of life in patients with transfusion-dependent thalassemia after hematopoietic SCT Bone Marrow Transplant 2008 42 5 319 327 10.1038/bmt.2008.165 18560410

[B32] Chiodi S Spinelli S Ravera G Petti AR Van Lint MT Lamparelli T Gualandi F Occhini D Mordini N Berisso G Quality of life in 244 recipients of allogeneic bone marrow transplantation Br J Haematol 2000 110 3 614 619 10.1046/j.1365-2141.2000.02053.x 10997973

[B33] Kasemsup V Schommer JC Cline RR Hadsall RS Citizen's preferences regarding principles to guide health-care allocation decisions in Thailand Value Health 2008 11 7 1194 1202 10.1111/j.1524-4733.2008.00321.x 18494755

